# Microwave Irradiation-Assisted Synthesis of a Novel Crown Ether Crosslinked Chitosan as a Chelating Agent for Heavy Metal Ions (M^+n^)

**DOI:** 10.3390/molecules15096257

**Published:** 2010-09-06

**Authors:** Awwad A. Radwan, Fars K. Alanazi, Ibrahim A. Alsarra

**Affiliations:** 1 College of Pharmacy, King Saud University, P.O. Box 2457, Riyadh 11451, Saudi Arabia; 2 Department of Pharmaceutical Organic Chemistry, Faculty of Pharmacy, Assiut University, Assiut-71527, Egypt; 3 Department of Pharmaceutics, College of Pharmacy, King Saud University, P.O. Box 2457, Riyadh 11451, Saudi Arabia; 4 Center of Excellence for Research in Engineering Materials, CEREM, Room 2A-138, College of Engineering, King Saud University, P. O. Box 800, Riyadh 11421, Saudi Arabia

**Keywords:** chitosan, crown ether, adsorption

## Abstract

Microwave irradiation was used to obtain a di-Schiff base type crosslinked chitosan dibenzocrown ether (CCdBE) via the reaction between the –NH_2_ and –CHO groups in chitosan and 4,4′-diformyldibenzo-18-c-6, respectively. The structure of the synthesized compound was characterized by elemental analysis, solid state ^13^C-NMR and FT-IR spectra analysis. The results showed that the mass fraction of nitrogen in the CCdBE derivative was much lower than those of chitosan. The FT-IR spectra of CCdBE revealed the expected chitosan-crown ether structure, as evidenced by the presence of the characteristic C=N and Ar peaks. The adsorption properties of CCdBE for Pd^2+^ and Hg^2+^ were investigated and the results demonstrated that the adsorbent has both desirable adsorption properties with a high particular adsorption selectivity for Hg^2+^ when in the presence of Pb^2+^ as well as selectivity coefficients for metal ions of *K*_Hg_^2+^_/Pb_^2+^ = 8.00 and *K*_Hg_^2+^_/Pb_^2+^ = 10.62 at pH values of 4 and 6, respectively. The reusability tests for CCdBE for Pb^2+^ adsorption showed that complete recovery of the ion was possible with CCdBE after 10-multiple reuses while CTS had no reusability at acidic solution because of its higher dissolution. The studied features of CCdBE suggested that the material could be considered as a new adsorbent. It is envisaged that the crosslinking of CTS into CCdBE would enhance practicality and effectiveness of adsorption in ion separation and removal procedures.

## 1. Introduction

Heavy metals are highly toxic at low concentrations and can accumulate in living organisms, causing several disorders and diseases [1,2]. As a result of industrialization and urbanization, the presence of heavy metal ions in water streams has increased greatly in the last fifty years. Removal of heavy metal ions from wastewater is essential because of their extreme environmental, public health and economic impacts [3]. 

The main techniques that have been used for metal content reduction from industrial waste are chemical precipitation, ion exchange, membrane filtration, electrolytic methods, reverse osmosis, solvent extraction, and adsorption [4,5,6]. However, these methods are limited by high operational costs and/or may also be inefficient in the removal of some toxic metal ions, particularly at trace level concentrations [7,8].

The use of chelation ion exchange for wastewater remediation has gained considerable attention in recent years. Chelation ion exchange, in contrast to simple ion exchange, has the advantage of only removing toxic metal ions while the harmless ions move on into the environment [9]. Some of the best chelation ion-exchange materials consist of different biopolymers and their derivatives because of the variety of functional groups, like –OH and –NH_2_, with which other chemical moieties, e.g., metal ions, can easily react and bond. These biopolymers, including cellulosics, alginates, proteins, chitin and chitin derivatives have remarkable capabilities of lowering metal ion concentrations to parts per billion levels [9,10]. For example, chitosan (CTS) is a deacetylated derivative of chitin that can adsorb metals due to its amino and hydroxyl groups. However, CTS can be dissolved in acidic media which limits its recycling in adsorption processes. Crosslinked chitosan synthesized by the reaction of CTS with hydrophobic crosslinking agents can overcome the disadvantages of CTS and still maintain good adsorption properties for many metal ions. Also, modifications to increase the number of binding sites and/or binding surfaces of chitosan have been made both by substitution on the amino group at C-2 or by crosslinking the polyglycans with small chemicals. Crosslinking CTS with biomass/biopolymers (e.g., alginate) [1,2], chelators such as ethylenediamine tetraacetic acid (EDTA) [11], fixatives such as glutaraldehyde (GA) [12] or polymers like polyvinyl alcohol (PVA) [13] creates a three-dimensional network within the biopolymer and increases the internal surface area for metal adsorption. Increase in structural and chemical stability of these crosslinked derivatives contributes to the resistance and endurance of acid [2] from surface and subsurface groundwater [14,15,16], thereby improving water/sewage purification treatments.

Because crown ethers have particular molecular structures, they have good and different complex selectivity for many metal ions. However, they are not recycled easily after use, so their applications are limited. If crown ethers were crosslinked to chitosan chains to give crown ether-crosslinked chitosan containing double structures and displaying the properties of chitosan and crown ethers, these novel chitosan derivatives would have stronger complex formation with better selectivity for metal ions than the corresponding crown ethers and chitosan separately [17]. 

**Scheme 1:**
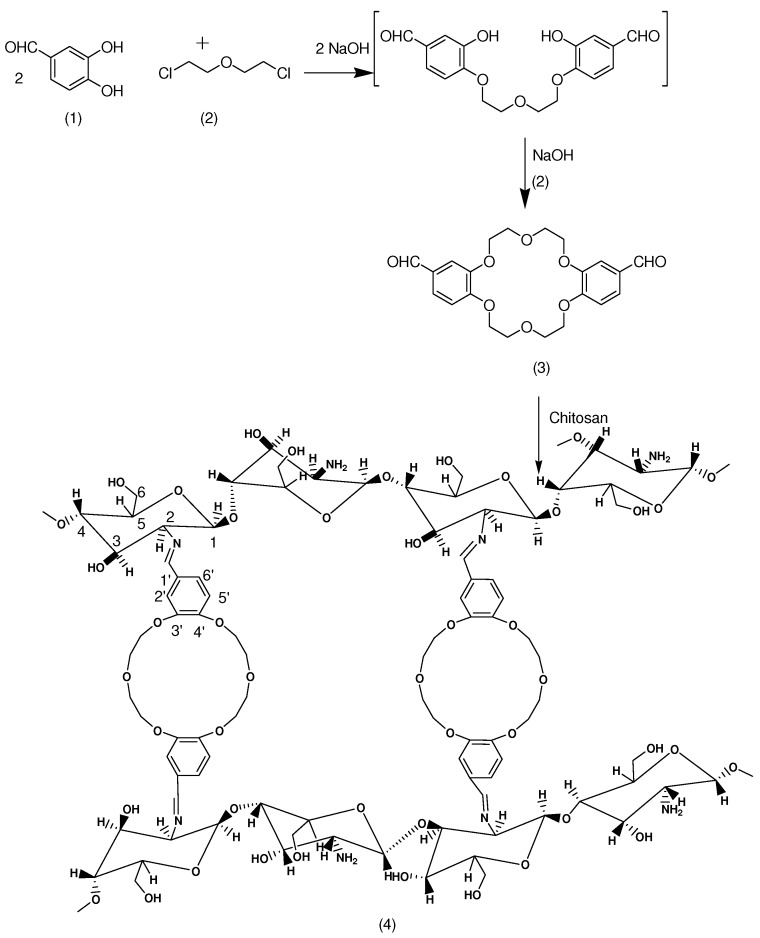
Reaction scheme for the synthesis of CcdBE.

Recently, microwave-assisted reactions have received much attention due to the higher conversions and shorter reaction times possible under microwave irradiation compared to those of conventional heating [18]. As a result, microwave irradiation as a chemical reaction means has been widely applied in various synthetic fields of chemistry such as organic and polymer synthesis [19]. In this work, we applied microwave technology to prepare a crosslinked chitosan dibenzocrown ether (CCdBE). This work thus serves as not only an expansion of the applications of microwave irradiation in reactions, but also as an expansion of the use of chitosan as an adsorbent for heavy metal removal. The chitosan derivative (CCdBE) was synthesized by the reaction of 4,4´-diformyldibenzo-18-crown-6 crown ether with CTS under microwave irradiation to give a *N*-Schiff base type crosslinked chitosan dibenzo-18-crown-6 (CCdBE) [20]. 4,4´-Diformyldibenzo-18-crown-6 was chosen because it was expected that the introduction of 4,4´-diformyldibenzocrown ether residues into chitosan could significantly enhance the adsorption ability compared with the parent CTS, making it possible for this CTS derivative to show promising applications in water treatment. 

The structure of the synthesized compound was confirmed by FT-IR spectra analysis, solid state ^13^C-NMR, elemental analysis and X-ray powder diffraction analysis. The adsorption of lead and mercury ions by CCdBE was studied and it showed that the cross linking of CTS into CCdBE could enhance the practicality and effectiveness of adsorption in ion separation and removal procedures.

## 2. Results and Discussion

### 2.1. Synthesis and Structural Characterization of Chitosan-Crown Ether

**Figure 1:**
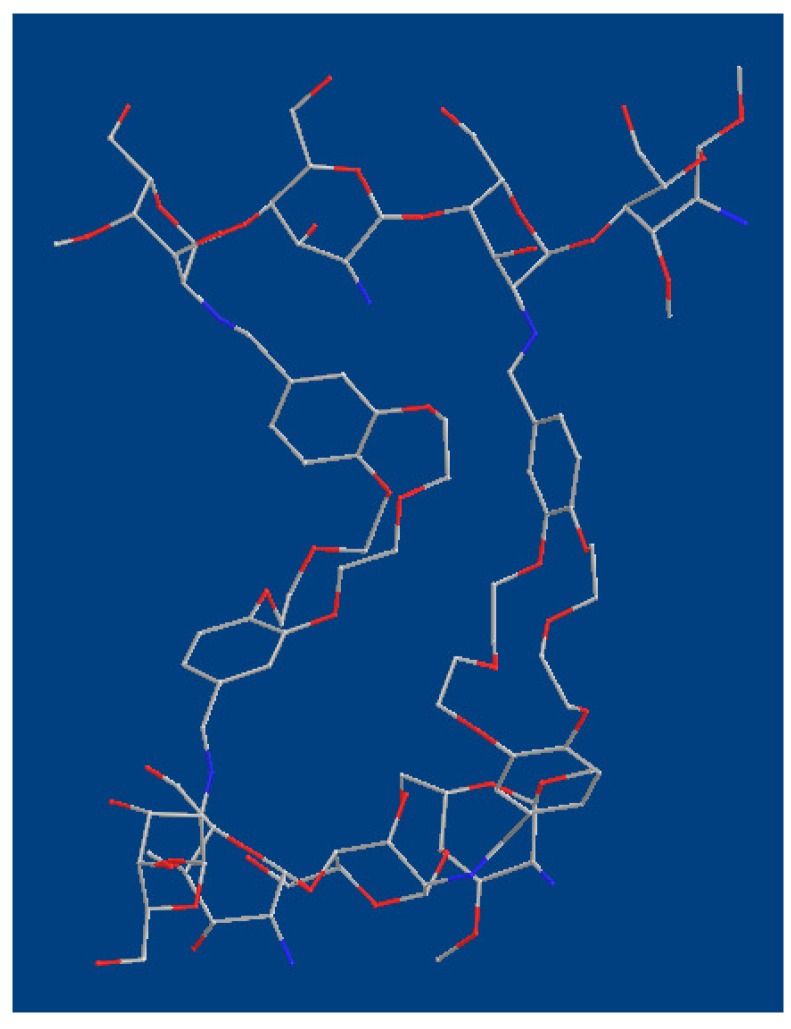
Three-dimensional (3D) view of CcdBE.

Chitosan crosslinked with dibenzo-18-crown-6 ether (CCdBE) was synthesized as shown in Scheme 1. The three-dimensional structure of the minimized conformation (Figure 1) of CCdBE showed that the crown ether rings were perpendicular to the crosslinked chitosan chains, which represent a series of parallel circular cavities passing over a cylindrical groove between the two crosslinked chitosan chains. 

Structure elucidation of crown ether crosslinked-chitosan (CCdBE) was accomplished by elemental analysis, FT-IR spectral analysis, X-ray diffraction analysis and solid state ^13^C-NMR analysis. The CCdBE derivative did not dissolve in organic solvents such as dimethylsulfoxide (DMSO), chloroform, formamide, dimethylformamide (DMF) and trifluoroacetic acid.

#### 2.1.1. Elemental Analysis

The elemental analysis results and degree of substitution (DS) of CTS and CCdBE are presented in Table 1. The nitrogen content of CCdBE was much lower than that of CTS. It was thought that the difference was attributable to the fact that the cross linker molecule (4´,4″-formyldibenzo-18-crown-6) does not have nitrogen atoms in its structure and although it had been grafted to chitosan by -CHO and -NH_2_ reaction, it only added carbon, oxygen and hydrogen atoms to CCdBE, decreasing the relative content of nitrogen in this molecule. Hydrogen content also decreased due to water elimination during the graft reaction and carbon content increased due to the high carbon content on cross linker molecule. 

**Table 1:** Elemental analysis of chitosan (CTS) and crosslinked chitosan (CCdBE).

Compound	N%	C%	H%	DS%
**CTS**	6.20	38.30	6.70	-
**CCdBE**	3.60	46.55	6.25	42

#### 2.1.2. Infrared Analysis

**Figure 2:**
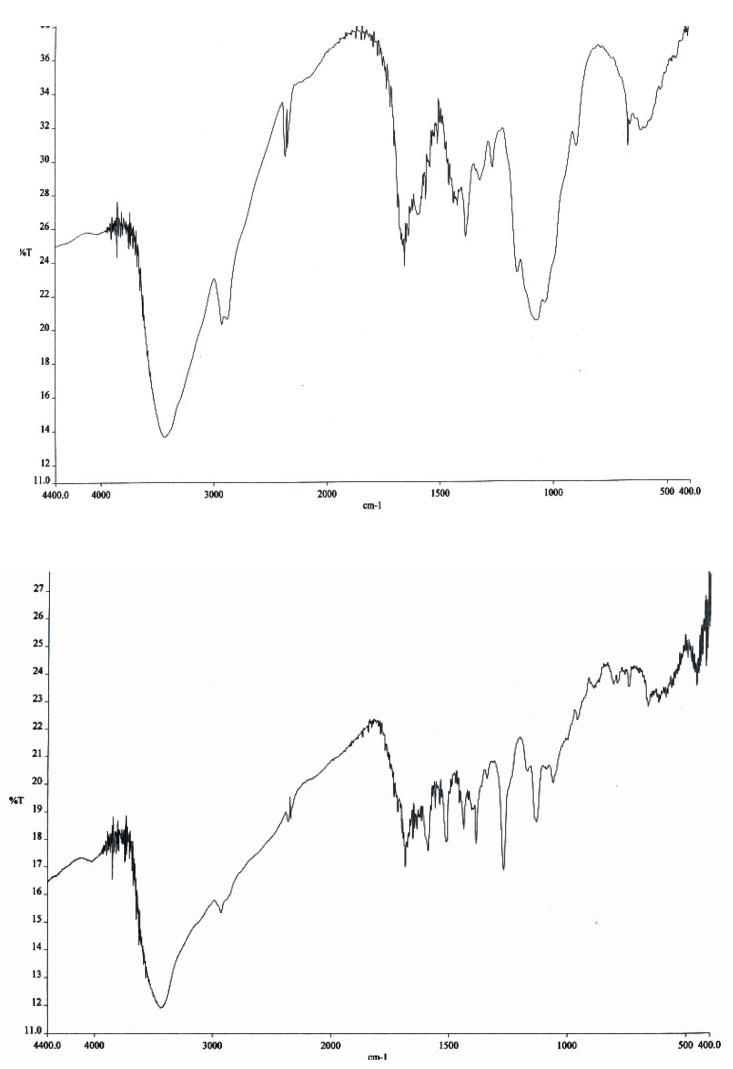
FTIR spectra of the chitosan (upper) and crosslinked chitosan (lower).

The IR spectra of chitosan and the derivative are shown in Figure 2. Although marked differences were not observed in the IR spectra, a characteristic C=N stretch vibration peak appeared at 1635 cm^-1^ due to the presence of the Schiff base groups produced in the course of the reaction (from chitosan to chitosan-crown ether). 

#### 2.1.3. X-ray Diffraction Analysis

**Figure 3:**
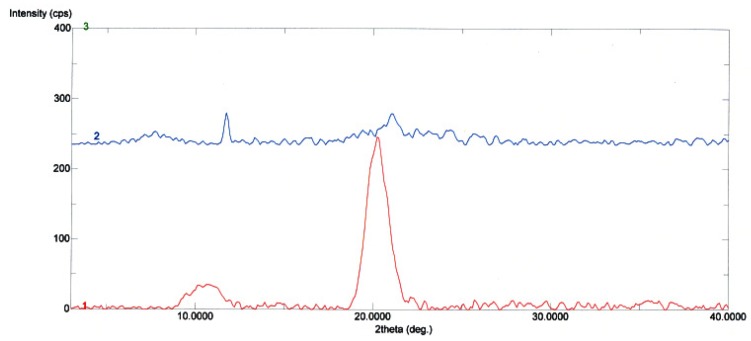
X-ray diffraction patterns of chitosan (in red) and crosslinked chitosan (blue).

The X-ray diffraction patterns of chitosan (CTS) and crosslinked chitosan (CCdBE) are shown in Figure 3. It shows the characteristic peaks of chitosan (CTS) at 2θ = 10º and 20º, which indicate a high degree of crystallinity for this substance [21,22]. The results revealed that the peak at 2θ = 10º disappeared and the peak at 2θ = 20º decreased in crosslinked chitosan (CCdBE). It appeared that CCdBE didn’t exhibit any crystallinity peak. It was thought that the decrease in crystallinity of CCdBE could be attributed to deformation of the strong hydrogen bond in the chitosan backbone chain because the amino groups were substituted by 4´,4″-diformyldibenzo-18-crown-6. The obtained results were in reasonable agreement with the published data of Xin-Hu *et al*. [23] who suggested that the crystallinity of chitosan was decreased when it is converted into a *N*-Schiff base-type benzo-21-crown-7 chitosan [23].

#### 2.1.4. Solid-State ^13^C-NMR Analysis

**Figure 4:**
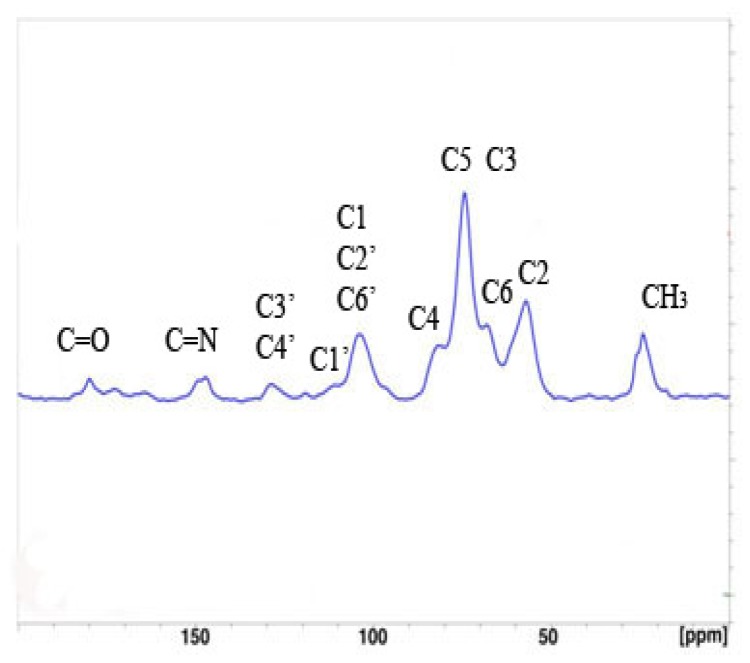
Solid-state ^13^C NMR spectrum of crosslinked chitosan (CCdBE).

The Solid-State ^13^C-NMR spectrum for crosslinked chitosan are shown in Figure 4. Characteristic aromatic carbon peak appeared at 127 ppm while the characteristic peak of carbon in C=N groups occurred at 148 ppm.

#### 2.1.5. Scanning Electron Microscopy (SEM) Studies

**Figure 5:**
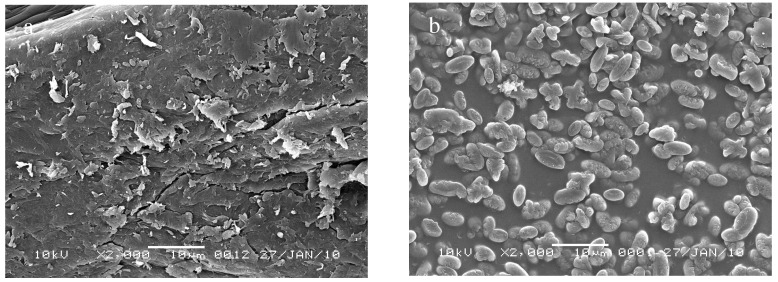
SEM of chitosan (a) and crosslinked chitosan (b).

Scanning electron microscope images of the surface of chitosan (CTS) and the crosslinked chitosan (CCdBE) are shown in Figure 5a,b. The surface morphology of chitosan (Figure 5a) was smooth, whereas the crosslinked chitosan (CCdBE, Figure 5b) exhibited a bean-like shaped granulated surface with high cavity-shaped porosities. The porous structure of (CCdBE) may be formed due to the bridging connection of diformyldibenzo-18-crown-6 molecules, hydrophobic in nature, between the different amine groups in CTS, hydrophilic surface, through the intra-molecular and/or inter-molecular crosslinking interactions that leads to deformation of the strong hydrogen bond in the chitosan backbone chain and resulted in an amorphous and a porous surface of CCdBE. The obtained results were confirmed using X-ray diffraction analysis. 

### 2.2. Adsorption Behavior

#### 2.2.1. Single Metal Ion Adsorption

The adsorption capacities for metal ions onto CTS and CCdBE are presented in Table 2. It was observed that the adsorption capacities of CCdBE for Pb^2+^ and Hg^2+^ were higher than that of CTS. This could be attributed to the three-dimensional network of CCdBE that provided a cavity tailored to the volumetric space of Pb^2+^ and Hg^2+ ^to adsorption. In addition, it can be seen that CCdBE has a better adsorption capacity for Hg^2+ ^which further suggest that CCdBE could selectively adsorb Hg^2+^. 

**Table 2:** Adsorption capacities of CTS and CCdBE for Pb^2+^ and Hg^2+ ^at pHs 4 and 6.

Adsorbent	Adsorption capacities (mmol/g)			
	pH 4		pH 6	
	Pb^2+^	Hg^2+^	Pb^2+^	Hg^2+^
CTS	0.82	0.33	0.94	1.48
CCdBE	0.99	1.10	1.18	1.58

#### 2.2.2. Two Metal Ion Co-Adsorption

The selective adsorption of CTS and CCdBE for Hg^2+^ and Pb^2+^ from their solution is shown in Table 3. By comparing the selectivity of CCdBE for Pb^2+^ and Hg^2+^ with that of CTS, it can be found that the adsorption of CCdBE for Hg^2+^ is greater than that for Pb^2+ ^at pHs 4 and 6, whereas the adsorption of Hg^2+ ^byCTS was slightly higher than that of Pb^2+ ^at pH 6 while the opposite occurred at pH 4. 

From the selectivity coefficient (k) results it can be observed that K_Hg/Pb_ has been improved 8-fold at pH 4 and 10.66-fold at pH 6 by crosslinking of CTS into CCdBE. The results further suggest that CCdBE can selectively recognize Hg^2+^. Furthermore, it is concluded that a selective separation of Pb^2+ ^and Hg^2+ ^could be affected by using the CCdBE.

**Table 3:** Adsorption selectivity of CTS and CCdBE for Pb(II) and Hg(II) ions.

Adsorbent	Adsorption capacities (mmol/g)		Selectivity coefficient (K_Hg_^2+^_/pb_^2+^)	Adsorption capacities (mmol/g)		Selectivity coefficient (K_Hg_^2+^_/pb_^2+^)
	pH 4			pH 6		
	Pb^2+^	Hg^2+^		Pb^2+^	Hg^2+^	
**CTS**	0.29	0.13	0.45	0.42	0.57	1.36
**CCdBE**	0.12	0.96	8.00	0.13	1.38	10.62

#### 2.2.3. Reusability

It was observed that the adsorption capacities of Pb^2+ ^decreased slightly from 1.2 to 1.08 mmol/g with increasing reuse cycles, and the adsorption capacity for Pb^2+^ is quite high even after reusing it 10 times, which indicates that the crosslinking in CCdBE results results in good reusability properties. 

## 3. Experimental Section

### 3.1. Materials

Chitosan of medium molecular weight with 75-85% deacetylation, 3,4-dihydroxybenzaldehyde bis(2-chloroethyl)ether and zinc chloride were purchased from Sigma-Aldrich Company (St. Louis, MO, USA). All aqueous solutions were prepared with deionized water that had been passed through a Millipore Milli-Q Plus water purification system (Millipore, Bedford, MA, USA). The metal salts chosen for this study ([PbCl_2_ and Hg(NO_3_)·0.5 H_2_O] were reagent grade. The compound 2,3,11,12-[4,4´-diformyl dibenzo]-1,4,7,10,13,16-hexaoxacyclooctadeca-2,11-diene (**3**) was synthesized using a previously reported procedure [20]. 

### 3.2. Synthesis of Crosslinked Chitosan diBenzocrown Ether (CCdBE) (4)

Powdered chitosan (1.13 g, 0.005 mol glucosamine residues) was dissolved in 20 mL of 10% (wt) acetic acid solution prepared in water to which ethanol (20 mL) was added and stirred using a magnetic stirrer for 30 min at 40 ºC. Then, 4´,4″-diformyldibenzo-18-c-6 crown ether (**3**), which was dissolved in chloroform-ethanol mixture (1:1, 5 mL) was slowly added dropwise into the above solution under nitrogen. The reaction mixture was irradiated in a microwave oven (Daewoo MW 800 W domestic type oven) at 10% intensity for 10 min. The reaction mixture was cooled and neutralized with sodium carbonate solution. The liquid was decanted from the reaction mixture. The remaining residue was washed with distilled water, and subsequently the aqueous layer was decanted. Washing and decantation were repeated twice with water and ethyl alcohol. The residue was dried under a reduced pressure overnight giving a pale brown fibrous solid (85% yield).

### 3.3. Structural Characterization of Chitosan-Crown Ether

#### 3.3.1. Elemental Analysis

The elemental analysis was performed on a Perkin Elmer CHNSO analyzer, model no. 2400 (Perkin Elmer, Inc., Waltham, MA, USA). 

#### 3.3.2. Infrared Spectra Analysis

IR analysis (Perkin Elmer FT-IR, Waltham, MA, USA) was employed to confirm the changes in functional groups for natural (CTS) and crosslinked chitosan (CCdBE). Infrared spectra (400-4000 cm^-1^) were recorded using 100-mg KBr discs containing 2% of chitosan (CTS) or crosslinked chitosan (CCdBE).

#### 3.3.3. X-ray Diffraction Analysis

The powder X-ray diffraction patterns of CTS and CCdBE were recorded with a Rigaku Ultima IV X-ray diffractometer (Osaka, Japan) using X-ray tube Cu (1.540562). Samples were irradiated with monochromatized Cu-Kα radiation and analyzed between 2θ angles of 5 and 40. The voltage, the current, and the time per step were 40 mV, 55 mA and 1s, respectively. Powder X-ray diffraction patterns were measured in order to evaluate the crystalline/amorphous character differences between CTS and CCdBE.

#### 3.3.4. Solid-state ^13^C-NMR Analysis

Solid state ^13^C-NMR spectra were recorded at the Center for Pharmaceutical Biotechnology (University of Illinois at Chicago, IL, USA) on a Bruker Avance operating at 500 MHz ^1^H frequency with a Bruker magic angle spinning probe with 4 mm diameter rotors. CPMAS spectra were collected at 9 kHz spinning speed and 55 kHz Two-Pulse Phase Modulation (TPPM) decoupling.

#### 3.3.5. Scanning Electron Microscopy (SEM) Studies

The shape and surface characteristics of CTS and CCdBE were observed using a scanning electron microscope (SEM). Samples were mounted on an aluminum stub using a double-sided adhesive carbon tape and the flakes were then sputter-coated with gold palladium (Au/Pd) using a vacuum evaporator (Edwards). The coated samples were then scanned and photomicrographs were taken with a Jeol (Tokyo, Japan) JSM1-5510 SEM instrument.

### 3.4. Adsorption Experiments

#### 3.4.1. Single Metal Ion Adsorption Experiment

A sample of CTS or CCdBE (25 mg) was added to a metal acetate solution (100.0 mL of 10 ppm initial M^2+^ concentration) with a given pH (pH 4 and 6) adjusted with 0.1 m/L acetic acid. The solution was shaken for 24 h at 25 ºC and then filtered. The adsorption capacities of metal ions were obtained from initial and final concentrations of metal ions in the acetate solution as determined by a Shimadzu atomic adsorption spectrophotometer, model AA-460-13 (Shimadzu Corporation, Tokyo, Japan).

#### 3.4.2. Two Metal Ion Co-Adsorption Experiment

A sample of CTS or CCdBE (25 mg) was added to acetate solution containing Pb^2+^ and Mg^2+ ^ions (100.0 mL, initial concentration of single species 5 ppm) at pH values of 4 and 6. The solution was shaken for 24 h at 25 ºC and then filtered. The contents of M^2+^ were determined from initial and final concentrations of metal ions in the acetate solution as determined by atomic adsorption spectrophotometry. 

#### 3.4.3. Reusability Experiment

The crosslinked chitosan (CCdBE) adsorbed Pb^2+ ^was dipped into stirring 0.1 mol/L HCl for 1 h at 25 ºC to remove Pb^2+^, and then treated with 0.1 mol/L NaOH for 5-8 h. Finally it was filtered and washed in turn with water, ethanol and ether. The CCdBE obtained was used in adsorption experiment and the process was repeated 10 times.

## 4. Conclusions

Microwave irradiation was utilized in the synthesis of a *N*-Schiff base-type crosslinked chitosan crown ether (CCdBE) by the reaction of 4´,4″-diformyldibenzo-18-crown-6 with chitosan. Its adsorption selectivity and reusability was determined. The adsorption capacity of the obtained CCdBE was much higher for Hg^2+^ than that for Pb^2+^. The reported crosslinking method could retain higher adsorption capacity of CTS and, at the same time, improve the acidic resistance of CTS with a desirable selectivity towards mercury ions over lead ions.
